# Satisfaction with caregivers during labour among low risk women in the Netherlands: the association with planned place of birth and transfer of care during labour

**DOI:** 10.1186/s12884-017-1410-9

**Published:** 2017-07-14

**Authors:** Caroline C. Geerts, Jeroen van Dillen, Trudy Klomp, Antoine L. M. Lagro-Janssen, Ank de Jonge

**Affiliations:** 1 Department of Midwifery Science, Academie Verloskunde Amsterdam Groningen (AVAG) and the EMGO Institute for Health and Care Research, VU University Medical Center Amsterdam, Van der Boechorststraat 7, 1081 BT Amsterdam, The Netherlands; 2 Department of Obstetrics and Gynaecology, Radboud University Medical Center Nijmegen, Geert Grooteplein Zuid 10, 6525 GA Nijmegen, the Netherlands; 3 Department of Women studies, Medical Sciences, Radboud University Medical Center Nijmegen, Geert Grooteplein Zuid 10, 6525 GA Nijmegen, the Netherlands

**Keywords:** Home childbirth, Transfer, Quality of care, Consumer Satisfaction, Caregiver, Midwifery

## Abstract

**Background:**

The caregiver has an important influence on women’s birth experiences. When transfer of care during labour is necessary, care is handed over from one caregiver to the other, and this might influence satisfaction with care. It is speculated that satisfaction with care is affected in particular for women who need to be transferred from home to hospital. We examined the level of satisfaction with the caregiver among women with planned home versus planned hospital birth in midwife-led care.

**Methods:**

We used data from the prospective multicentre DELIVER (Data EersteLIjns VERloskunde) cohort-study, conducted in 2009 and 2010 in the Netherlands. Women filled in a postpartum questionnaire which contained elements of the Consumer Quality index. This instrument measures *'general rate of* *satisfaction with the caregiver’* (scale from 1 to 10, with cut-off of below 9) and *‘quality of treatment by the caregiver*’ (containing 7 items on a 4 point Likert scale, with cut-off of mean of 4 or lower).

**Results:**

Women who planned a home birth (*n* = 1372) significantly more often rated *'quality of treatment by caregiver'* high than women who planned a hospital birth (*n* = 829). Primiparous women who planned a home birth significantly more often had a high rate (9 or 10) for ‘*general satisfaction with caregiver’* (adj.OR 1.48; 95% CI 1.1, 2.0). Also, primiparous women who planned a home birth and had care transferred during labour (331/553; 60%) significantly more often had a high rate (9 or 10) for ‘*general satisfaction’* compared to those who planned a hospital birth and who had care transferred (1.44; 1.0–2.1). Furthermore, they significantly more often rated ‘*quality of treatment by caregiver’* high, than 276/414 (67%) primiparous women who planned a hospital birth and who had care transferred (1.65; 1.2–2.3). No differences were observed for multiparous women who had planned home or hospital birth and who had care transferred.

**Conclusions:**

Planning home birth is associated to a good experience of quality of care by the caregiver. Transferred planned home birth compared to a transferred planned hospital birth does not lead to a more negative experience of care received from the caregiver.

**Electronic supplementary material:**

The online version of this article (doi:10.1186/s12884-017-1410-9) contains supplementary material, which is available to authorized users.

## Background

Previously it was found that a substantial proportion of Dutch women look back negatively on their birth experience [1]. A negative description of caregivers and transfer of care from midwife-led primary to obstetrician-led secondary care during labour, were both associated with negative feelings about the birth experience three years postpartum. A negative birth experience is related to several negative (health) outcomes [2–6].

To monitor women’s experiences, studies have obtained information on childbirth experience [7], and women’s satisfaction with the care they received by care providers during labour [8]. Both outcomes are indicators of quality of care, and they are mutually related. Factors that are important to childbirth satisfaction, are based on the relationship with, and care provided by the caregiver [9]. In a thematic analysis of 62 studies, the relationship with the caregiver and support were identified as key attributes of the childbirth experience [9]. Therefore, the focus of the current study is on quality of care provided by caregivers which we will refer to as ‘satisfaction with the caregiver’.

In case of transfer of care, multiple caregivers are involved in the labour process. It is important to gain insight in how the experience with the caregivers is affected when care is transferred. Especially, since referral rates in the Netherlands are rising and therefore an increasing number of women are affected by the consequences of transfer of care. During labour referral rates have risen for primiparous women from 50% in 2008 to 63% in 2014 and from 17% to 26% for multiparous women [10, 11]. Caregivers have the potential to make an important difference to women’s experience when transfer of care is necessary [12].

In the Netherlands, low-risk women receive midwife-led care from primary care midwives. These are women with a singleton pregnancy of a fetus in cephalic presentation who do not have any medical or obstetric risk factors that are an indication for obstetrician-led care, and who start labour between 37 and 42 weeks. If complications or obstetric risk factors occur during pregnancy, labour, or after birth, women have care transferred from midwife-led to obstetrician-led care. In obstetrician-led care women may receive care from clinical midwives, obstetric registrars, and obstetric nurses, under the final responsibility of an obstetrician or by obstetricians themselves. After transfer, the primary care midwife may provide support to women, but this does not happen in all cases. The indications for transfer of care are layed out in the obstetric indication list (VIL) [13]. Women who still receive midwife-led care at term can choose to give birth at home or in hospital, assisted by their primary care midwife.

Recently it was suggested that transfer of care during labour affects patient satisfaction particularly among women who plan home birth [14]. It is speculated that transportation from home to hospital during labour might contribute to this. Additionally expectations (giving birth at home) are not met.

The primary aim of this study was to evaluate satisfaction with the caregiver among women who planned a home or a hospital birth in midwife-led care. Secondly, we studied the effect of transfer of care in a planned home birth compared to transfer of care in a planned hospital birth on satisfaction with the caregiver.

## Methods

### DELIVER-study

The Deliver study is a multicenter prospective cohort study into the quality, organisation and accessibility of midwifery care in the Netherlands, which was described extensively elsewhere [15].

Briefly, the means of recruitment of clients was through midwifery practices. Purposive sampling was used to select practices, using three stratification criteria: region (north, centre, south), level of urbanisation (urban or rural area), and practice type (dual or group practice). Twenty of the 519 midwifery practices across the Netherlands participated in this study. Between September 2009 and December 2010 client data were collected using questionnaires. Clients who received antenatal care and who gave informed consent, were given up to three questionnaires: one before 34 weeks gestation (the 1st questionnaire), one between 34 weeks gestation and birth (the 2nd questionnaire), one approximately 6 weeks postpartum (the 3rd questionnaire). The response rate of the DELIVER study was 62%.

The Deliver client data were linked to primary midwife-led care data from the Netherlands Perinatal Register (PRN, “Landelijke Verloskundige Registratie”, LVR1). Linkage was successful in 76.1% of the women included in this study.

For the women with successful linkage, agreement between LVR1 and Deliver data for women who started labour in midwife-led care was 99.1% for vacuum or forceps extraction, 99.9% for caesarean section and 99.4 (hospital) to 94.7% (home) for actual place of birth. In case of disagreement, we used data from the DELIVER study.

### Study population

For this study, participants with singleton term pregnancies that were in midwifery care at the onset of labour were selected. The definition for onset of labour in primary care is based on information from the LVR1 database. This information showed internal inconsistencies in 3.7% of the cases. Women who had care transferred for prolonged rupture of membranes (>24 h without contractions) were excluded. Among these women, transfer to secondary care occurred before start of the dilation (first) stage, and thus planned place of birth is unlikely to have affected satisfaction with the caregiver. Women who were transferred to secondary care during pregnancy and women who were advised to give birth in hospital in midwife led care because of a condition that would increase the risk of complications for the woman or baby were also excluded.

### Planned place of birth and transfer of care during labour

Planned place of birth (home or hospital under midwife-led care) is recorded on the LVR-1 form at some point during pregnancy. This information is missing for some women; midwives may forget to record the details or the women may not have made a decision on where to give birth until the onset of labour. The number of women with unknown planned place of birth in this study will be reported in the Results section.

When complications arise such as listed in the VIL [13], care is transferred from midwife-led to obstetrician-led care. When a woman is at home, this requires transport to a hospital facility prior to transfer of care, either by car, or if transport by car is too inconvenient or in case of an emergency, by ambulance. In this study, both transfer during labour or immediately postpartum, were defined as transfer of care.

From information on planned and actual place of birth two groups were formed of women who were transferred, according to their planned place of birth. Three groups were formed for women who gave birth in primary care (and thus were not transferred), see Table 1:

**Table 1:** Definition of planned place of birth and transfer

Planned home birth:	Birth planned at home in midwife-led care.
Planned hospital birth:	Birth planned in hospital under midwife-led care.
Transfer:	Women who had care transferred from midwife-led to obstetrician-led during labour or immediately postpartum.
Home – transfer:	planned home birth but transfer to obstetrician-led care during labour or immediately postpartum.
Hosp - transfer:	planned hospital birth but transferred to secondary care during labour or immediately postpartum.
No transfer:	Women who gave birth in midwife-led care without being transferred.
Home - home:	birth planned at home and actual birth at home under midwife-led care
Hosp- hosp:	planned hospital, actual hospital birth in midwife-led care
Hosp-home:	planned hospital birth in midwife-led care, but actual home birth in midwife-led care

In the analyses for the primary research question planned hospital birth (including hosp-transferred and hosp-hosp) was the comparison group, because the hypothesis was that quality of treatment and general satisfaction with the caregiver is negatively influenced by transportation from home to hospital when transfer of care is necessary.

### Satisfaction with caregiver

Satisfaction with the caregiver during labour was assessed with the 3rd postpartum DELIVER client questionnaire, which was filled in 6 weeks postpartum on average (see Additional file 1).

Satisfaction of care by caregiver was assessed using 1: general and 2: evaluative questions which both were adapted from the Consumer Quality Index (CQ-index) for maternity care. This instrument was developed to measure client experiences. The questions were identified and validated in item- and factor analyses [8, 16]. The cut-off values for both outcomes were chosen based on the fact that self-reported satisfaction with care is usually very high [17], particularly in relation to maternity care [18]. We aimed to identify whether a planned home or hospital birth is associated to rates that indicate anything less than very good care.

1. ‘*General ratings of satisfaction’* with care provided by caregiver during labour ranged from 1 = worst possible care, to 10 = best possible care. The rate was dichotomised into ‘below 9’, and ‘equal to and above 9’ to distinguish between very high and (somewhat) lower level of satisfaction with care.
2. The evaluative questions, measuring ‘*quality of treatment by a particular caregiver’* during labour, consisted of 7 items: feeling in safe hands, having things explained in an understandable way, being treated with respect, being listened to carefully, being taken seriously, being given enough time, being given enough opportunity to ask questions. Questions were framed as: “Did you experience….?” with answers ranging from 1 = never, to 4 = always. The mean of the total score of the 7 items was calculated, ranging from 1 to 4 [8]. We used a cut-off of ‘below 4’ and ‘equal to 4’. The internal consistency of the 7 items was high, with a Cronbach’s alpha of 0.93.

The cut-off of 9 was chosen, based on a previous study showing that ‘*general satisfaction with caregiver’* during labour and birth was rated 9 or 10 in more than half of the women. The cut-off for *‘quality of treatment’* was set on 4 vs lower than 4 (1, 2, 3), based on the high mean score of this variable in the previous study (e.g. 3.61 to 3.92) [8].

### Confounding factors

Maternal age, ethnic background (Dutch, western background or non-western background) and social status (score 1–4) were taken into account, because of their association with planned place of birth [19, 20] and satisfaction with childbirth [21]. For social status we used the score developed by the Netherlands Institute for Social Research (SCP), using postal codes, based on education, income and employment rates. A low score equals high social status (www.scp.nl/Onderzoek/Lopend_onderzoek/A_Z_alle_lopende_onderzoeken/Statusscores).

Results were presented separately by parity, because the association between planned place of birth and satisfaction with the caregiver might differ for primi- and multiparous women. Once a woman has given birth, her choice about place of birth is influenced by her previous experience [22], and multiparous women are more satisfied with the birth experience and thus might rate satisfaction with care differently [21].

### Potential explanatory factors

Some factors were assessed to explore if they could explain associations between planned place of birth and satisfaction with the caregiver. Some of these factors are only applicable to women in obstetrician-led care. However, both among the women who planned home birth as well as among the women who planned hospital birth in midwife-led care, transfer during labour to obstetrician-led care occurred in some cases, and consequently some women who planned home or hospital birth in midwife-led care received medical pain relief during labour for example.

Medical pain relief (yes-no) and experience of labour pain (rating scale from 1 to 10) were taken into account, because they have been reported to influence satisfaction with childbirth and consequently might influence satisfaction with the caregiver [21]. Management and experience of labour pain is different for women planning hospital birth, compared to women planning a home birth. The role of medical interventions, including augmentation, vaginal instrumental delivery and caesarean section was investigated [21]. Finally, in an explanatory analysis the extent to which transfer of care (yes/no) could explain possible differences in the association between planned place of birth and satisfaction with the caregiver was assessed.

### Data-analysis

Baseline and pregnancy related characteristics and labour outcomes were compared between low risk women who planned to give birth at home versus women who planned to give birth in hospital using student’s t-test for continuous and chi-square test for categorical characteristics. Mean scores of ‘*general satisfaction with the caregiver’* and ‘*quality of treatment’* were calculated for planned home and hospital birth, to calculate the minimal important difference (MID = *0.2* * SD_hospital_) [23, 24]. It is a score that reflect differences in a client derived outcome of an intervention, that is meaningful to the client. The association between planned place of birth (home/ hospital) and satisfaction with the caregiver during labour (defined as 1: ‘*general rating of satisfaction with the caregiver’* and, 2: the ‘*quality of treatment by the caregiver’*) was analysed using multilevel logistic regression analysis with two levels; the midwifery practice level and individual level, in order to account for clustering of women within midwifery practices. In similar models the association between planned place of birth and the 7 separate evaluative questions for ‘*quality of treatment by the caregiver’* were analysed, as well as the association between planned place of birth and transfer (home-transfer, hosp-transfer) or not (home-home, hosp-home, hosp-hosp) with *‘general satisfaction with the caregiver*’ and ‘*quality of treatment’*. To examine the effect of transfer in a planned home birth on satisfaction with the caregiver, as well as the overall effect of satisfaction in a planned home birth compared to a planned hospital birth, planned hospital was used as the reference.

Odds ratios and 95% confidence intervals were presented. Dummy variables were constructed in case of >2 categories. Adjustments were made for confounders using multivariable models. Additionally, potential explanatory factors were added separately in the models. Finally, a sensitivity analysis was performed including women with and without discrepancies in the definition for start of labour in primary care.

All analyses were stratified for parity. The analysis were performed using SPSS 20.0 and Stata 10. Statistical significance was considered with a *p*-value <0.05.

## Results

**Fig. 1:**
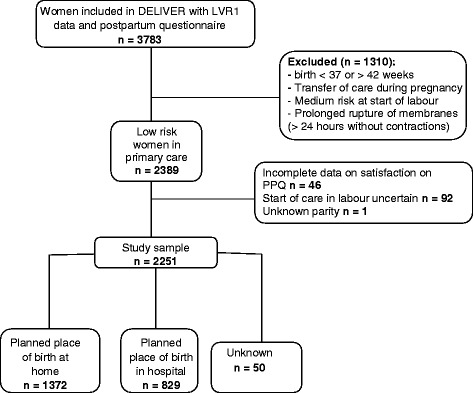
Selection of low risk women who started labour in primary care in the Deliver study

In the DELIVER dataset, 6021 women had data linked to LVR1. In Fig. 1 is it shown that data from the postpartum questionnaire (PPQ) were available for 3783 of these women. Of these, 1394 women were excluded because of a medium risk indication, prolonged rupture of membranes without contractions, preterm or postterm birth or transfer of care during pregnancy. Of the remaining 2389 women, 92 were excluded because of discrepancies in the definition for onset of labour in midwife-led care, 46 had incomplete data about satisfaction with the caregiver on the PPQ and 1 had unknown parity. Of the remaining 2251 eligible women, 1372 women planned a home birth (61%) and 829 (37%) women planned a hospital birth. Planned place of birth was unknown in 50 women (2%).

Furthermore, 826 women had care transferred during labour either intrapartum (*n* = 726) or immediately postpartum (*n* = 99).

Table 2 shows that women who chose to give birth at home were significantly less likely to be primiparous, of ethnic minority background and below 25 years or above 35 years, compared to women who chose hospital birth. Women planning a home birth significantly less often had augmentation or were transferred to secondary care and they had a significant lower rate of instrumental vaginal delivery, caesarean section, pharmacological pain relief and experience of labour pain. Both the mean score for ‘*general satisfaction with the caregiver’* and ‘*quality of treatment’* were significantly higher for women who planned home birth. The MID for ‘*general rate of satisfaction’* was 0.26 (0.2*1.3) and for ‘*quality of treatment’* 0.08 (0.2*0.4).

**Table 2:** Baseline and pregnancy related characteristics and labour outcomes for planned place of birth of women in the primary care setting at the start of labour

	Planned home birth	Planned hospital birth	*p*-value
	*n* = 1329	*n* = 829	
Baseline characteristics			
Parity, n (%)			< 0.001
Primiparous	577 (42.1)	414 (49.9)	
Multiparous	795 (57.9)	415 (50.1)	
Gestational age, n (%)			0.19
37 weeks	38 (2.8)	33 (4.0)	
38–40 weeks	1064 (77.6)	648 (77.6)	
41 weeks	270 (19.7)	148 (17.9)	
Maternal age, n (%)			0.02
< 25 years	105 (7.7)	74 (9.3)	
25–35 years	1040 (75.8)	583 (70.3)	
> 35 years	227 (16.5)	172 (20.7)	
Ethnic background, n (%)			< 0.001
Dutch	1244 (90.9)	645 (78.1)	
Western background	74 (5.4)	87 (10.5)	
Non-western background	51 (3.7)	94 (11.4)	
Socioeconomic position, n (%)			0.57
1st tertile	353 (25.8)	228 (27.7)	
2nd tertile	645 (47.2)	386 (46.8)	
3rd tertile	369 (27.0)	210 (25.5)	
Maternal outcomes and interventions			
Experience of labour pain, mean (sd)			
first stage	7.2 (2.0)	7.7 (1.9)	< 0.001
second stage	6.9 (2.2)	7.2 (2.2)	0.001
General rating of care by caregiver, mean (sd)	9.1 (1.1)	8.9 (1.3)	0.001
Quality of treatment, mean (sd)	3.9 (0.3)	3.8 (0.4)	< 0.001
Transfer during labour, n (% referred)	435 (31.7)	390 (47.0)	< 0.001
Medical pain relief^a^, n (% yes)	141 (10.3)	184 (22.4)	< 0.001
Instrumental/ operative delivery, n (%)			0.02
Vacuum−/ forceps extraction	123 (9.0)	99 (12.0)	
Secondary caesarean section	48 (3.5)	39 (4.7)	
Augmentation, n (% yes)	205 (14.9)	171 (20.7)	0.001
Neonatal outcomes			
Apgar score < 7, n (%)	8 (0.6)	7 (0.8)	0.47
Complications directly postpartum, n (%)	21 (1.6)	14 (1.8)	0.72

Missings: maternal age *n* = 1; ethnic background *n* = 10; socioeconomic position *n* = 10; general rating of care by caregiver *n* = 28; CQ index score *n* = 2

^a^Medicinal pain relief includes epidural (193), remiphentanyl (87) and opioids (86)

### Planned place of birth and satisfaction with the caregiver

Table 3 shows that for primiparous women during labour both the 1: ‘*general rate of satisfaction’* with the caregiver, and 2: ‘*quality of treatment’*, were significantly more often high among women planning a home birth compared to women planning a hospital birth, taking into account confounders and clustering of women within midwifery practices. For multiparous women, 2: ‘*quality of treatment’* was statistically significantly more often high (equal to 4) among women who planned a home birth.

**Table 3:** Planned place of birth and 1: *general rate of satisfaction with the caregiver *(<9/ ≥ 9) and 2: *quality of treatment by caregiver *during labour and birth (below 4/equal to 4), by parity

	Primiparous women				Multiparous women			
	Multilevel				Multilevel			
1: General rating of satisfaction with caregiver	Total N	N (%) ≥ 9	OR (95% CI)	Adjusted^a^ OR (95% CI)	Total N	N (%) ≥ 9	OR (95% CI)	Adjusted^a^ OR (95% CI)
Planned home	575	408 (71.0)	1.50 (1.1, 2.0)**	1.48 (1.1, 2.0)**	788	625 (79.3)	1.07 (0.8, 1.4)	1.08 (0.8, 1.5)
Planned hospital	404	249 (61.6)	1	1	406	317 (78.1)	1	1
2: Quality of treatment by caregiver		N (%) =4				N (%) =4		
Planned home	576	428 (74.3)	1.74 (1.3, 2.3)**	1.79 (1.3, 2.4)**	795	631 (79.4)	1.65 (1.3, 2.2)**	1.58 (1.2, 2.1)**
Planned hospital	413	260 (63.0)	1	1	413	289 (70.0)	1	1

**p < 0.05

^a^adjusted for maternal age, ethnic background (Dutch, western background, non-western background) and socioeconomic status (quartiles)

The separate items of the measure for 2: ‘*quality of treatment by the caregiver’* in Table 4 shows that primi- and multiparous women who planned a home birth significantly more often had the feeling that they were ‘in safe hands with the caregiver’ and that the caregiver ‘took them seriously’, compared to women who planned a hospital birth. In addition, nulliparous women who planned a home birth significantly more often stated that ‘the caregiver explained things in an understandable way’. Multiparous women who planned a home birth significantly more often experienced that ‘the caregiver listened to them carefully’.

**Table 4:** Satisfaction with caregiver concerning different items of *quality of treatment* (below 4/equal to 4) in relation with planned place of birth of women under midwife led care at the start of labour

	Primiparous women				Multiparous women			
			Crude	Adjusted^a^			Crude	Adjusted^a^
	Total	No (%) always (=4)	OR (95% CI)	OR (95% CI)	Total	No (%) always (=4)	OR (95% CI)	OR (95% CI)
Did you feel in safe hands with the caregiver?								
planned home	577	497 (86.1)	1.74 (1.2, 2.5)**	1.57 (1.1, 2.3)**	795	700 (88.1)	1.56 (1.1, 2.2)**	1.46 (1.04, 2.1)**
planned hospital	414	323 (78.0)	1	1	413	341 (82.6)	1	1
Did the caregiver explain things in an understandable way?								
planned home	576	514 (89.2)	1.91 (1.3, 2.8)**	1.93 (1.3, 2.9)**	795	718 (90.3)	1.43 (1.0, 2.1)	1.32 (0.9, 1.9)
planned hospital	414	336 (81.2)	1	1	413	358 (86.7)	1	1
Did the caregiver treat you with respect?								
planned home	577	544 (94.3)	1.36 (0.8, 2.4)	1.27 (0.7, 2.3)	795	751 (94.5)	1.46 (0.9, 2.4)	1.36 (0.8, 2.2)
planned hospital	414	382 (92.3)	1	1	413	380 (92.0)	1	1
Did the caregiver listen to you carefully?								
planned home	576	510 (88.5)	1.42 (0.97, 2.1)	1.46 (0.99, 2.1)	795	720 (90.6)	1.68 (1.2, 2.4)**	1.57 (1.1, 2.3)**
planned hospital	414	350 (84.5)	1	1	413	351 (85.0)	1	1
Did the caregiver take you seriously?								
planned home	576	519 (90.1)	1.57 (1.1, 2.3)**	1.54 (1.0, 2.3)**	795	741 (93.2)	1.91 (1.3, 2.9)**	1.76 (1.2, 2.7)**
planned hospital	414	354 (85.5)	1	1	413	361 (87.4)	1	1
Did the caregiver spent sufficient time with you?								
planned home	576	488 (84.7)	1,49 (1.1, 2.1)**	1,41 (0.99, 2.0)	795	700 (88.1)	1.34 (0.9, 1.9)	1.28 (0.9, 1.8)
planned hospital	413	325 (78.7)	1	1	413	347 (84.0)	1	1
Did the caregiver give you enough opportunity to ask questions?								
planned home	576	527 (91.5)	1,51 (0.99, 2.3)	1,41 (0.9, 2.2)	795	746 (93.8)	1.48 (0.9, 2.3)	1.35 (0.9, 2.2)
planned hospital	414	363 (87.7)	1	1	413	375 (90.8)	1	1

**p < 0.05

^a^adjusted for maternal age, ethnic background (Dutch, western background, non-western background) and socioeconomic status (quartiles)

### Explanatory analysis

For primiparous women, the significantly lower ‘*general rating of satisfaction with the caregiver’* during labour for women who planned hospital birth, was no longer present after adjustment for medical pain relief (data not shown in table): adjusted OR 1.31 (95% CI 0.95–1.80). Since women who planned hospital birth more often received medical pain relief, the results of the explanatory analyses suggest that satisfaction with the caregiver was lower for women who received medical pain relief and that this partly explains why women who planned hospital birth in midwife-led care rated satisfaction with caregiver lower. Another explanation was provided by transfer of care: the association was abolished when adjusting for transfer of care during labour: adjusted OR 1.33 (0.96–1.83). Adjustment for medical interventions (e.g. vaginal instrumental delivery, caesarean section and augmentation) or experience of labour pain did not have an effect on the associations.

### Transfer and satisfaction with caregiver

Compared to women who planned a hospital birth and who were transferred during labour as the reference group (hosp-transfer), ‘*general rating of satisfaction with care’* and ‘*quality of treatment by the caregiver’* during labour was significantly more often high for primiparous women who planned a home birth and who were transferred (home-transfer; Table 5). No differences were observed in any measure of satisfaction with care for multiparous women who planned a home or hospital birth and who were transferred (Table 5; transfer).

**Table 5:** Odds ratios for the association between 1: *general rate of satisfaction with the caregiver* (<9/ ≥ 9) and 2: *quality of treatment by caregiver* during labour and birth (below 4/ equal to 4) among women who were transferred (transfer) during labour and among those who were not transferred (no transfer)

Primiparous women					Multiparous women			
			Multilevel				Multilevel	
1: General rating of satisfaction with caregiver	Total N	N (%) ≥ 9	OR (95%CI)	Adjusted^a^ OR (95% CI)	Total N	N (%) ≥ 9	OR (95%CI)	Adjusted^a^ OR (95% CI)
Transfer								
planned home-transfer	329	208 (63.2)	1.53 (1.1, 2.2)**	1.44 (1.0, 2.1)**	104	70 (67.3)	0.97 (0.5, 1.7)	1.11 (0.6, 2.1)
planned hosp-transfer	267	139 (52.1)	1	1	109	74 (67.9)	1	1
No transfer								
planned home-home	222	181 (81.5)	1.41 (0.7, 2.7)	1.48 (0.8, 2.9)	652	529 (81.1)	1.21 (0.8, 1.8)	1.21 (0.8, 1.8)
planned hosp-home	39	34 (87.2)	2.14 (0.7, 6.4)	2.04 (0.7, 6.2)	100	89 (89.0)	2.28 (1.1, 4.7)**	2.31 (1.1, 4.7)**
planned hosp-hosp	98	76 (77.6)	1	1	197	154 (78.2)	1	1
2: Quality of treatment by caregiver		N (%) =4				N (%) =4		
Transfer								
Planned home	330	225 (68.2)	1.61 (1.2, 2.2)**	1.65 (1.2, 2.3)**	104	78 (75.0)	1.72 (0.9, 3.1)	1.76 (0.9, 3.3)
Planned hospital	275	157 (57.1)	1	1	113	72 (63.7)	1	1
No transfer								
planned home-home	222	185 (83.3)	1.73 (0.9, 3.2)	1.81 (0.9, 3.4)	659	530 (80.4)	1.87 (1.3, 2.7)**	1.80 (1.3, 2,6)**
planned hosp-home	39	29 (74.3)	1.01 (0.4, 2.4)	0.94 (0.4, 2.3)	101	80 (79.2)	1.74 (0.9, 3.1)	1.70 (0.9, 3.0)
planned hosp-hosp	99	74 (74.7)	1	1	199	137 (68.8)	1	1

**p < 0.05

^a^adjusted for maternal age, ethnic background (Dutch, western background, non-western background) and socioeconomic status (quartiles)

Table 5, no transfer, shows that multiparous women who planned a hospital birth but who actually gave birth at home (hosp-home) significantly more often gave a higher rate for ‘*general satisfaction with the caregiver’* than multiparous women who planned a hospital birth and who actually gave birth in hospital in midwife-led care (hosp-hosp). Multiparous women who planned and had a birth at home (home-home) significantly more often rated ‘*quality of treatment of care’* high compared to women who planned a hospital birth and who actually gave birth in hospital in midwife-led care (hosp-hosp).

The group who planned a home birth but actually gave birth in hospital in midwife-led care was too small for analysis.

### Sensitivity analysis

Sensitivity analysis of 2286 low-risk women (the eligible study population including 85 women with discrepancies in the definition of start of labour in midwife-led care and known planned place of birth) for the association of planned place of birth and 1: ‘*general rating of satisfaction with the caregiver’* and 2: ‘*quality of treatment of care’*, showed similar results.

## Discussion

### Main findings

Primiparous women in midwife-led care at the start of labour who planned a home birth were significantly more satisfied with the care they received from the caregiver during labour than women who planned a hospital birth. They rated ‘*quality of treatment provided by the caregiver’* significantly higher when they had care transferred compared to primiparous women who planned a hospital birth and who had care transferred. Multiparous women who planned a home birth were significantly more satisfied with ‘*quality of treatment’* and when they had care transferred they were equally satisfied with the care provided by the caregiver compared to multiparous women who planned a hospital birth and who had care transferred during labour.

### Strengths and limitations

For this study, we used data from a prospective cohort study. Although a randomised controlled trial would be preferred for planned place of birth this was shown not to be feasible [25]. Therefore, due to the observational nature of this study, we adjusted the association for confounders. In addition, clustering of women within midwifery practices was taken into account. Data were not complete for all eligible women, including data on planned place of birth. Some women do not choose their place of birth until they are in labour and in some cases the midwife might have forgotten to register this information. ‘*Quality of treatment’* and ‘*general rating of care’* by caregiver was not available for all women. Furthermore, women in the DELIVER study were higher educated and less often of non-Dutch origin than the general Dutch population [15]. In addition, the response rate for participation was only 62%. It seems, however, unlikely that among non-responders, the association between planned place of birth and quality of care would be in the opposite direction. In this study we used the CQ-index. This instrument has been validated among Dutch pregnant women [8]. A reliability analysis of the 7-items measuring ‘*quality of treatment by the caregiver’* revealed a high internal consistency as well. However, measuring satisfaction or quality of treatment by the caregiver is a methodological challenge, and therefore we have interpreted the findings in the scope of clinical relevance in addition to statistical significance as described below.

### Interpretation

To our knowledge, our study is the first to report on the association between planned place of birth and satisfaction with caregiver among women who were transferred during labour. It was shown previously that women who (planned to or actually) give birth at home have higher scores on ‘*general satisfaction’* or ‘*quality of care’* received from the caregiver [8, 26]. Furthermore it has been reported that women who planned a home birth were less satisfied with the caregiver and with their birth experience when they were transferred from home to hospital, compared to those who had a home birth and were not transferred [27, 28]. These studies, however, did not compare women who had transfer of care during labour and who planned birth at home versus in hospital. Another study found that transfer of care affected evaluation of the midwife similarly among women with a planned home birth compared to those with a planned hospital birth [22]. In our study primiparous women who planned a home birth and who were transferred during labour were more satisfied with the caregiver compared to primiparous women who planned a hospital birth and who were transferred. In multiparous women the results were in the same direction, although not statistically significant.

It is remarkable that our findings are not consistent with the assumption that transport and not meeting expectation would negatively influence satisfaction with caregivers among women who planned home birth [14]. Notably, women who plan hospital birth need to be transported during labour at some point as well. Furthermore, women who opt for a home birth have fundamental trust in their independent ability to give birth and have a trust in the birth process in general [20, 29, 30]. Possibly, this difference in motives might cause women to rate satisfaction with the caregiver differently, e.g. ‘feeling in safe hands with the caregiver’. Medical pain relief resulted in fewer women rating satisfaction with the caregiver highly among primiparous women. This partly explained a higher rating of satisfaction among women who plan home birth, since they less often receive medical pain relief. Possibly women who receive pain medication have had a longer and more exhausting labour which might affect satisfaction with caregiver.

Nevertheless, planned place of birth itself may also have some effect on satisfaction with the birth experience. Satisfaction with the caregiver differed between two different planned birth environments (home or hospital in midwife-led care), even after adjustment for confounders. It may be that midwives behave differently when they provide care in hospital than in a woman’s home. Another study showed an association between environment (e.g. ward design) and behaviour of care providers [31]. In our study, primi- and multiparous women more often stated that the caregiver took them seriously, or listened to them carefully (only multiparous women), or explained things in an understandable way (only primiparous women). This confirms reports that showed that environmental changes can influence the quality of interaction with the patient [31, 32]. Hospital rooms that were associated with improved patient outcomes were those that offered privacy, promoted social support and were calming [33]. It seems likely that the home environment satisfies these aspects more often than a hospital environment.

The cut-off point for satisfaction differentiated between very satisfied versus less than very satisfied. The majority of women were satisfied with the caregiver most of the time. Overall, the mean difference in both ratings of ‘*general satisfaction’* and ‘*quality of treatment’* of women who planned a home birth compared to women who planned a hospital birth was smaller than the minimally important difference for clinical relevance [23, 24].

Apart from health outcomes, experiences of women have become increasingly important aspects of good quality care. Previous studies in the Netherlands found no association between planned home birth and severe adverse maternal and neonatal health outcomes [19, 34, 35]. Recently, we showed that feelings of control were similar among women with planned home birth and who were transferred to the hospital, compared to women with planned hospital birth who were transferred [36]. Therefore, it seems justified that women in the Netherlands are offered a choice for place of birth, either at home or hospital.

## Conclusion

The current study shows that planned home birth among low risk women does not lead to reduced satisfaction with caregiver compared to planned hospital birth. In addition, a transferred planned home birth compared to a transferred planned hospital birth does not lead to a more negative experience of care received from the caregiver.

The findings in this study can help women to make a well informed choice about the place of birth. Women should be informed that when transfer of care during labour occurs, satisfaction with the caregiver is similar when planning either a home or a hospital birth. Future studies should aim to gain more insight in how care during transfer could be optimized in the Netherlands and countries with a similar maternity care system.

## Additional file

Additional file 1: Third postpartum questionnaire. This questionnaire was filled in by women after birth. The questionnaire contains questions including the CQ-index (see question 54) which measures quality of treatment by caregiver, and a general rate of satisfaction with care (see question 60). (PDF 134 kb)

## Abbreviations

CQ-index: Consumer Quality Index
LVR1: “Landelijke Verloskundige Registratie” for primary care
MID: Minimal Important Difference
PRN: Netherlands Perinatal Register
SCP: Netherlands Institute for Social Research
VIL: “Verloskundige Indicatie Lijst”
