# Supplementary material for: Satisfaction with caregivers during labour among low risk women in the Netherlands: the association with planned place of birth and transfer of care during labour
Source: BMC Pregnancy Childbirth. 2017 Jul 14;17:229. doi: 10.1186/s12884-017-1410-9 (PMC5513372; doi:10.1186/s12884-017-1410-9)
Supplement: Additional file 1: — Third postpartum questionnaire. This questionnaire was filled in by women after birth. The questionnaire contains questions including the CQ-index (see question 54) which measures quality of treatment by caregiver, and a general rate of satisfaction with care (see question 60). (PDF 134 kb) [file 12884_2017_1410_MOESM1_ESM.pdf]

Deliver.
data eerstelijns verloskunde

Deze vragenlijst start met een aantal vragen over uzelf en uw contact(en) met de verloskundige praktijk.

1. Bent u op dit moment **zwanger**?
0 nee
0 ja, momenteel ………. weken (afronden op hele weken) → *u hoeft deze vragenlijst niet verder in te vullen en kunt hem nu opsturen.*

2. Bent u onlangs **bevallen** (een miskraam valt hier in dit geval niet onder)?
0 ja → *ga door naar vraag **6.***
0 nee

3. Wat was de **reden** voor uw contact met de verloskundige praktijk:
0 kinderwensgesprek → *u hoeft deze vragenlijst niet verder in te vullen en kunt hem nu opsturen.*
0 ik was zwanger maar heb een miskraam gehad → *ga door naar de volgende vraag*
0 ik was zwanger maar heb de zwangerschap af laten breken → *u hoeft deze vragenlijst niet verder in te vullen en kunt hem nu opsturen.*
0 anders, namelijk ……………………………………………………. → *u hoeft deze vragenlijst niet verder in te vullen en kunt hem nu opsturen.*

Een miskraam is een zeer trieste gebeurtenis. We begrijpen dan ook dat het lastig kan zijn om nog enkele vragen te beantwoorden. Toch willen we uw medewerking vragen om uw mening te geven over de aan u gegeven zorg, want ook in geval van een miskraam verdient u goede zorg.

4. **Hoe vaak** heeft u contact gehad met de verloskundige?
0 1 maal
0 2-3 maal
0 Meer dan 3 maal

5. Door wie werd u **begeleid** in de periode rondom uw miskraam? *(meerdere antwoorden mogelijk)*
0 Huisarts
0 Verloskundige
0 Gynaecoloog
0 Anders, namelijk ....
0 Niemand
→ *U hoeft deze vragenlijst niet verder in te vullen en kunt hem nu opsturen.*

Hieronder volgt een aantal vragen over de bevalling.

6. Werd de bevalling **begeleid** door één of meer van de volgende personen?
(*meerdere antwoorden mogelijk)*
0 een verloskundige of waarneemster uit de eigen praktijk
0 de huisarts
0 de gynaecoloog of arts-assistent in het ziekenhuis
0 verloskundige van het ziekenhuis of geboortecentrum
0 een verloskundige in opleiding
0 anders, namelijk ………………………
0 niemand

7. Met hoeveel **weken** zwangerschap bent u bevallen?
0 vóór 37 weken zwangerschap
0 met 37 weken
0 met 38 weken
0 met 39 weken
0 met 40 weken
0 met 41 weken
0 met 42 weken
0 na meer dan 42 weken zwangerschap

8. Op welke **datum** is uw kind **geboren**:
Geboortedatum kind: ……………………………

9. Op welk **tijdstip** van de dag is uw kind geboren:
24-uurs notatie
geboortetijdstip kind: …………uur……...minuut

10. Was dit 's ochtends, 's middags, 's avonds of 's nachts?
0 's Ochtends
0 's middags
0 's avonds
0 's nachts

11. **Waar** vond de bevalling plaats?
0 thuis
0 thuis, maar ik moest zelf na de geboorte naar het ziekenhuis
0 thuis, maar de baby moest na de geboorte naar het ziekenhuis
0 thuis begonnen, maar uiteindelijke geboorte in het ziekenhuis
0 op eigen verzoek in het ziekenhuis of geboortecentrum
0 om medische redenen in het ziekenhuis
0 elders, namelijk ………………………………….

12. Waren er, naast zorgverleners, nog **anderen aanwezig** bij de bevalling?
(*meerdere antwoorden mogelijk)*
0 geen anderen aanwezig
0 mijn partner
0 (schoon)moeder/-ouders
0 kind(eren)
0 vriend(in)
0 anderen, namelijk ………………………………………….

13. Hoe is de bevalling **verlopen**?
(*meerdere antwoorden mogelijk)*
0 geheel spontaan en zonder ingrepen
0 ik ben ingeknipt
0 de placenta kwam niet vanzelf, moest verwijderd worden
0 de vliezen zijn door een zorgverlener gebroken om de bevalling op gang te brengen
0 de bevalling is in het ziekenhuis ingeleid
0 de weeën zijn kunstmatig (door middel van een infuus) versterkt
0 ik ben na de bevalling gehecht
0 de baby is met een kunstverlossing (met tang of vacuümpomp) geboren
0 het is een keizersnede geworden
0 het was een geplande keizersnede, voordat de bevalling op gang kwam ⟶ *ga door naar vraag **21.***

14. Heeft u behoefte gehad aan een vorm van **pijnbestrijding**? (*dit kan zijn met of zonder medicijnen*)
0 ja
0 nee

15. Heeft u gebruik gemaakt van een behandeling van de pijn tijdens de bevalling **zonder medicijnen**?
0 nee → *ga door naar vraag **17.***
0 ja → Wat voor soort behandeling heeft u gehad? (*meerdere antwoorden mogelijk)*
0 rugmassage
0 warm bad of douche
0 drukpuntmassage
0 geboorte-Tens (apparaatje waarmee u zich kleine stroomstootjes toedient via plakkertjes op uw rug)
0 acupunctuur
0 anders, namelijk ………………………………………………………………….

16. Was dat de behandeling van de pijn die u graag wilde?
0 ja
0 nee

17. Heeft u gebruik gemaakt van een vorm van het tegengaan van de pijn **met medicijnen** tijdens de bevalling?
0 nee → *ga door naar vraag **19.***
0 ja → Wat voor soort behandeling heeft u gehad? (*meerdere antwoorden mogelijk)*
0 injectie(s) (spuiten) met pijnstilling
0 infuus met een pompje waarbij u zelf de dosering kunt bepalen
0 ruggenprik (eventueel met doseerpompje die u zelf kon regelen)
0 algehele verdoving
0 anders, namelijk ………………………………………………………………………

18. Was dat de behandeling van de pijn die u graag wilde?
0 ja
0 nee

19. Hoeveel pijn heeft u ervaren tijdens de **ontsluitingsweeën**?
Geef dit aan door een getal te omcirkelen op onderstaande schaal, waarbij 1 staat voor helemaal geen pijn en 10 voor de ergst mogelijke pijn.

Helemaal geen pijn — De ergst mogelijke pijn
1 1½ 2 2½ 3 3½ 4 4½ 5 5½ 6 6½ 7 7½ 8 8½ 9 9½ 10
O Niet van toepassing (geen weeën gehad)

20. Hoeveel pijn heeft u ervaren tijdens de **persweeën**?
Geef dit aan door een getal te omcirkelen op onderstaande schaal, waarbij 1 staat voor helemaal geen pijn en 10 voor de ergst mogelijke pijn.

Helemaal geen pijn — De ergst mogelijke pijn
1 1½ 2 2½ 3 3½ 4 4½ 5 5½ 6 6½ 7 7½ 8 8½ 9 9½ 10
O Niet van toepassing (geen weeën gehad)

21. Was dit uw eerste bevalling?
0 Ja → *ga door naar vraag **24.***
0 Nee

22. Hoeveel pijn heeft u ervaren tijdens de **ontsluitingsweeën** van uw vorige bevalling?
Geef dit aan door een getal te omcirkelen op onderstaande schaal, waarbij 1 staat voor helemaal geen pijn en 10 voor de ergst mogelijke pijn.

Helemaal geen pijn — De ergst mogelijke pijn
1 1½ 2 2½ 3 3½ 4 4½ 5 5½ 6 6½ 7 7½ 8 8½ 9 9½ 10
O Niet van toepassing (geen ontsluitingsweeën gehad)

23. Hoeveel pijn heeft u ervaren tijdens de **persweeën** van uw vorige bevalling?
Geef dit aan door een getal te omcirkelen op onderstaande schaal, waarbij 1 staat voor helemaal geen pijn en 10 voor de ergst mogelijke pijn.

Helemaal geen pijn — De ergst mogelijke pijn
1 1½ 2 2½ 3 3½ 4 4½ 5 5½ 6 6½ 7 7½ 8 8½ 9 9½ 10
O Niet van toepassing (geen persweeën gehad)

24. **Welke vormen** van het tegengaan van de pijn tijdens de bevalling heeft uw verloskundige met u besproken, voordat u ging bevallen? (*meerdere antwoorden mogelijk)*
0 rugmassage
0 warm bad of douche
0 drukpuntmassage
0 geboorte-Tens (apparaatje waarmee u zich kleine stroomstootjes toedient via plakkertjes op uw rug)
0 acupunctuur
0 injectie(s) (spuiten) met pijnstilling
0 infuus met een pompje waarbij u zelf de dosering kunt bepalen
0 ruggenprik
0 anders, namelijk …………………………
0 geen

25. Had u voorafgaand aan de bevalling een voorkeur voor een bepaalde manier van het tegengaan van de pijn tijdens de bevalling?
0 nee → *ga door naar vraag 27*
0 ja

26. Is uw voorkeur voor het tegengaan van de pijn tijdens de bevalling **vastgelegd** in uw dossier of geboorteplan?
0 ja
0 nee
0 weet niet

*Indien u een geplande keizersnede heeft gehad, dan kunt u vraag **27** t/m **29** overslaan en doorgaan met vraag **30**.*

27. Kon u zich tijdens de weeën nog steeds bewegen zoals u graag wilde (lopen, zitten, liggen, een bad of douche nemen)?
Indien u meerdere bevallingen heeft gehad, betreft dit de laatste bevalling
0 ja
0 nee → Waarom niet? *(meerdere antwoorden mogelijk)*
0 vanwege de medische apparatuur moest ik op bed blijven
0 vanwege de pijnmedicatie kon ik mijn benen niet meer goed bewegen
0 vanwege de pijnmedicatie was ik niet meer helder
0 er werd mij gezegd dat het voor mij beter was om op bed te blijven
0 de zorgverlener had liever dat ik op bed bleef zodat zij/hij er beter bij kon
0 de weeën waren te pijnlijk en/of te snel achter elkaar
0 anders, namelijk ………………………………………………………………

*Indien u geen ontsluitingsweeën heeft gehad, dan kunt u vraag **28** overslaan.*

28. Wilt u voor het volgende blok met uitspraken aangeven aan hoe zich voelde tijdens de **ontsluitingsweeën** voorafgaande aan het persen?

	De hele tijd of bijna de hele tijd	Ongeveer driekwart van de tijd	Iets meer dan de helft van de tijd	Ongeveer de helft van de tijd	Iets minder dan de helft van de tijd	Ongeveer een kwart van de tijd	Niet of bijna niet
Ik voelde me gespannen	0	0	0	0	0	0	0
Ik voelde me belangrijk	0	0	0	0	0	0	0
Ik had zelfvertrouwen	0	0	0	0	0	0	0
Ik had het gevoel mezelf in de hand te hebben	0	0	0	0	0	0	0
Ik was bang	0	0	0	0	0	0	0
Ik voelde me ontspannen	0	0	0	0	0	0	0
Ik had het gevoel dat ik het goed deed	0	0	0	0	0	0	0
Ik voelde me hulpeloos	0	0	0	0	0	0	0
Ik voelde me machteloos	0	0	0	0	0	0	0
Ik had het gevoel bij de mensen te zijn die om me geven	0	0	0	0	0	0	0
Ik had het gevoel te falen	0	0	0	0	0	0	0

*Indien u geen persweeën/uitdrijvingsweeën heeft gehad, dan kunt u vraag **29** overslaan.*

29. Wilt u voor het volgende blok met uitspraken terugdenken aan hoe u zich voelde tijdens **het persen ofwel de uitdrijvingsweeën**?

	De hele tijd of bijna de hele tijd	Ongeveer driekwart van de tijd	Iets meer dan de helft van de tijd	Ongeveer de helft van de tijd	Iets minder dan de helft van de tijd	Ongeveer een kwart van de tijd	Niet of bijna niet
Ik voelde me gespannen	0	0	0	0	0	0	0
Ik voelde me belangrijk	0	0	0	0	0	0	0
Ik had zelfvertrouwen	0	0	0	0	0	0	0
Ik had het gevoel mezelf in de hand te hebben	0	0	0	0	0	0	0
Ik was bang	0	0	0	0	0	0	0
Ik voelde me ontspannen	0	0	0	0	0	0	0
Ik had het gevoel dat ik het goed deed	0	0	0	0	0	0	0
Ik voelde me hulpeloos	0	0	0	0	0	0	0
Ik voelde me machteloos	0	0	0	0	0	0	0
Ik had het gevoel bij de mensen te zijn die om me geven	0	0	0	0	0	0	0
Ik had het gevoel te falen	0	0	0	0	0	0	0

30. In welke **houding** heeft u uw kind gebaard?
0 zittend in bed of op baarkruk
0 zittend in bad
0 hurkend, of op handen en voeten
0 liggend op een bed of operatietafel
0 anders, namelijk ……………………………………..
0 weet ik niet

31. Heeft u binnen een uur na de geboorte **huidcontact** met uw baby gehad?
0 ja
0 weet ik niet
0 nee ⟶ Waarom niet?
0 vanwege complicaties bij de baby
0 vanwege complicaties bij mij zelf (bijvoorbeeld keizersnede)
0 anders, namelijk ……………………………………………………..…..

32. Heeft u binnen een uur na de geboorte de gelegenheid gekregen om met **borstvoeding** te beginnen?
0 ja
0 nee ⟶ Waarom niet?
0 vanwege complicaties bij de baby
0 vanwege complicaties bij mij zelf (bijvoorbeeld keizersnede)
0 ik had aangegeven dat ik geen borstvoeding wilde geven
0 anders, namelijk ……………….. ……………………………………..
0 weet niet

ZORGGEBRUIK

We willen u graag twee vragen stellen over een deel van de verloskundige zorg tijdens uw zwangerschap.

33. Heeft u tijdens de zwangerschap één of meer van de onderstaande **onderzoeken** naar de gezondheid van uw kind laten uitvoeren?
(meerdere antwoorden mogelijk)
0 nee, ik heb geen onderzoek laten uitvoeren
0 termijnecho (echo bij ongeveer 12 weken)
0 combinatie test (screening op het Downsyndroom)
0 20 weken echo
0 vruchtwaterpunctie
0 vlokkentest
0 anders, namelijk ....

34. Hoe **kijkt u achteraf terug** op uw keuze om dergelijk onderzoek wel of niet uit te laten voeren?
0 ik zou achteraf gezien dezelfde keuze weer maken
0 ik zou achteraf gezien een andere keuze maken, namelijk, …………………………………………
0 weet ik niet

De kraamperiode is meestal een leuke tijd vol nieuwe dingen, maar kan ook vermoeiend zijn waardoor jonge moeders wel wat hulp kunnen gebruiken. We willen u nu enkele vragen stellen over uw gebruik van en ervaringen met de kraamzorg.

*Indien u een geplande keizersnede heeft gehad, dan kunt u vraag **35** t/m **38** overslaan en doorgaan met vraag **39**.*

35. Was er een **kraamverzorgende** aanwezig bij de bevalling?
0 nee ⟶ *ga door naar vraag **39.***
0 ja

36. Op welk moment was de kraamverzorgende **gearriveerd**?
0 ruim vóór de geboorte van de baby
0 kort voor of tijdens de geboorte van de baby
0 na de geboorte van de baby

37. **Hoe lang** was de kraamverzorgende aanwezig tijdens de bevalling?
0 minder dan 1 uur
0 meer dan 1 uur maar minder dan 2 uur
0 meer dan 2 uur

38. Is de kraamverzorgende **na de bevalling gebleven** om die dag kraamzorg te verlenen?
0 ja, ze is gebleven
0 nee, ze is na de bevalling weggegaan
0 weet ik niet

39. Heeft u professionele kraamzorg **thuis** gehad?
0 nee → *ga door naar vraag **48.***
0 ja

40. **Waarvoor** heeft u die kraamzorg thuis gehad?
(meerdere antwoorden mogelijk)
0 hulp tijdens de bevalling (spullen klaarzetten, opruimen, wassen, enz.)
0 emotionele steun tijdens de bevalling
0 hulp bij verzorging van mijn baby (wassen, baden, aankleden, voeden, enz.)
0 hulp bij verzorging van mezelf (wondverzorging, temperatuur, enz.)
0 voorlichting, advies en instructie over borstvoeding en flesvoeding
0 voorlichting, advies en instructie over babyverzorging, slaap/waakritme baby, slaaphouding enz.
0 voorlichting, advies en instructie over uw eigen verzorging, rust, hygiëne, enz.
0 hulp bij huishoudelijk werk (koken, boodschappen, de was, schoonmaken enz.)
0 hulp bij verzorging gezin (zorg voor overige kinderen, kraamvisite, enz.)
0 anders, namelijk ………………………………………………………………………………………

41. Op **hoeveel dagen** na de bevalling heeft u thuis kraamzorg gehad?
…………………. dagen

42. **Hoe lang** was de kraamverzorgende dagelijks (ongeveer) aanwezig?
0 minder dan 3 uur
0 meer dan 3 uur maar minder dan 5 uur
0 meer dan 5 uur

43. Was de kraamverzorgende dagelijks (ongeveer) **even lang** aanwezig?
0 ja
0 nee, de eerste dagen was ze langer aanwezig dan de laatste dagen
0 nee, de eerste dagen was ze korter aanwezig dan de laatste dagen
0 weet ik niet

44. Heeft u het aantal uren kraamzorg gekregen dat voor de bevalling met u was **afgesproken**?
0 ja → *ga door naar vraag **46.***
0 nee, ik heb minder uren kraamzorg gekregen dan was afgesproken → *ga door naar de volgende vraag*
0 nee, ik heb meer uren kraamzorg gekregen dan was afgesproken → *ga door naar de volgende vraag*
0 weet ik niet → *ga door naar vraag **46.***

45. Wat is de **reden** dat u niet het afgesproken aantal uren kraamzorg heeft gekregen?
(meerdere antwoorden mogelijk)
0 verblijf in het ziekenhuis na de bevalling
0 meerling
0 conditie baby
0 conditie moeder
0 problemen met voeding
0 kosten (eigen bijdrage) te hoog
0 eigen verzoek
0 te weinig personeel bij de kraamorganisatie
0 anders, namelijk……………………

46. Wat vindt u van het **aantal uren** kraamzorg dat u heeft gehad?
0 te weinig
0 net voldoende
0 ruim voldoende
0 te veel

47. Heeft u steeds **dezelfde kraamverzorgende** in huis gehad?
0 ja
0 ja, alleen de kraamverzorgende die bij de bevalling was, was een andere dan die daarna kwam
0 nee, na een paar dagen kwam een andere kraamverzorgende
0 nee, er zijn drie of meer verschillende kraamverzorgenden bij mij thuis geweest

48. Heeft uw baby in de eerste week een **hielprik** gehad?
0 nee
0 weet ik niet
0 ja → Door wie is de hielprik uitgevoerd?
0 verloskundige
0 huisarts
0 kraamverzorgende
0 wijkverpleegkundige
0 verpleegkundige/laborante in het ziekenhuis
0 weet ik niet

49. Heeft uw baby een **gehoortest** gehad?
0 nee → *ga door naar vraag **51.***
0 ik weet niet meer of mijn baby een gehoortest heeft gehad → *ga door naar vraag **51***
0 ja, in de eerste week na de geboorte
0 ja, na de eerste week
0 ja, maar ik weet niet meer wanneer

50. Door wie is de gehoortest uitgevoerd?
0 verloskundige
0 huisarts
0 kraamverzorgende
0 wijkverpleegkundige
0 verpleegkundige/laborante in het ziekenhuis
0 weet ik niet

51. Heeft u een **nacontrole** bij uw verloskundige of gynaecoloog gehad?
0 Ja, bij de verloskundige
0 Ja, bij de gynaecoloog
0 Ik heb een afspraak gemaakt, maar ben nog niet geweest
0 Nee → Waarom niet?
0 Geen behoefte aan / ik vind het niet nodig
0 Alles gaat goed
0 Ik ben te kort geleden bevallen
0 Anders, namelijk ……………………………………………

52. Hoeveel **weken oud** is uw kindje nu? (op het moment van invullen van de vragenlijst).
0 jonger dan 2 weken
0 vanaf 2 weken tot 4 weken
0 vanaf 4 weken tot en met 6 weken
0 ouder dan 6 weken

53. Geeft u op dit moment **borstvoeding**?
0 Ja
0 Gedeeltelijk, in combinatie met flesvoeding
0 Nee, alleen flesvoeding

ERVARING MET DE ZORGVERLENING

Tijdens en na de bevalling heeft u waarschijnlijk contact gehad met een verloskundige en kraamverzorgende. Om de verloskundige zorg en kraamzorg te kunnen verbeteren, is het belangrijk om de mening van vrouwen te weten over deze zorg. Hieronder volgt een aantal vragen over uw ervaringen met uw verloskundige zorgverlener (verloskundige uit uw eigen praktijk, huisarts, verloskundige uit het ziekenhuis of gynaecoloog) en kraamzorg tijdens de bevalling en de kraamperiode.

54. Wilt u voor de volgende vragen aangeven hoe vaak dit **tijdens de bevalling** het geval was?

	**Nooit**	**Soms**	**Meestal**	**Altijd**
Had u het gevoel bij de verloskundige zorgverlener in vertrouwde handen te zijn geweest?	0	0	0	0
Legde de verloskundige zorgverlener dingen op een begrijpelijke manier uit?	0	0	0	0
Behandelde de verloskundige zorgverlener u beleefd?	0	0	0	0
Luisterde de verloskundige zorgverlener aandachtig naar u?	0	0	0	0
Nam de verloskundige zorgverlener u serieus?	0	0	0	0
Besteedde de verloskundige zorgverlener voldoende tijd aan u?	0	0	0	0
Kon u de verloskundige zorgverlener vragen stellen als u iets wilde weten?	0	0	0	0

55.

	Nederland	Indonesië / voormalig Nederlands-Indië	Suriname	Marokko	Turkije	Nederlandse Antillen of Aruba	Anders, namelijk
In welk land bent u geboren?	0	0	0	0	0	0	…………
In welk land is uw moeder geboren?	0	0	0	0	0	0	…………
In welk land is uw vader geboren?	0	0	0	0	0	0	…………

56. Welke **taal** spreekt u meestal thuis?
0 Nederlands
0 Fries
0 Nederlands dialect
0 Engels
0 Surinaams
0 Marokkaans
0 Turks
0 Arabisch
0 Berbers
0 Anders, namelijk:……………………..

*Indien u of een van uw ouders in het buitenland is geboren, beantwoord dan graag de volgende vraag (**57**).*

57.

	Nooit	Soms	Meestal	Altijd	Niet van toepassing
Had de verloskundige zorgverlener begrip voor thuisgewoontes rond de geboorte, die anders zijn dan bij autochtone Nederlanders?	0	0	0	0	0

*Iedereen graag de volgende vraag beantwoorden (**58**).*

58. Wat zijn uw **ervaringen** met uw verloskundige zorgverlener?

De verloskundige zorgverlener die mij begeleidt …	Nooit	Soms	Meestal	Altijd
… behandelt iedereen gelijkwaardig ongeacht geloof, afkomst of huidskleur (discrimineert niet)	0	0	0	0
… heeft belangstelling voor mijn cultuur en achtergrond	0	0	0	0
… heeft kennis van gebruiken rond bevalling en kraambed in andere culturen	0	0	0	0

*Indien u thuis meestal een andere taal spreekt dan Nederlands, Fries, of Nederlands dialect, beantwoord dan graag vraag **59**.*

59. Wat zijn uw **ervaringen** met uw verloskundige zorgverlener?

De verloskundige zorgverlener die mij begeleidt …	Nooit	Soms	Meestal	Altijd
… heeft er begrip voor als ik, door taalproblemen, soms moeilijk mijn verhaal kan vertellen	0	0	0	0
… heeft voorlichtingsfolders in mijn eigen taal	0	0	0	0
… schakelt, als ik dat op prijs stel, een tolk in	0	0	0	0

60. Wilt u hieronder het cijfer **omcirkelen** dat het best uw **totaaloordeel** weergeeft over de zorg door uw verloskundige zorgverlener **tijdens de bevalling**?
1 betekent: de slechtst mogelijke zorg en 10 betekent: de best mogelijke zorg

De slechtst mogelijke zorg — De best mogelijke zorg
1 2 3 4 5 6 7 8 9 10

61. Wilt u voor de volgende vragen aangeven hoe vaak dit **tijdens de kraamperiode** het geval was?

	Nooit	Soms	Meestal	Altijd
Had u het gevoel bij de verloskundige zorgverlener in vertrouwde handen te zijn geweest?	0	0	0	0
Legde de verloskundige zorgverlener dingen op een begrijpelijke manier uit?	0	0	0	0
Behandelde de verloskundige zorgverlener u beleefd?	0	0	0	0
Luisterde de verloskundige zorgverlener aandachtig naar u?	0	0	0	0
Nam de verloskundige zorgverlener u serieus?	0	0	0	0
Besteedde de verloskundige zorgverlener voldoende tijd aan u?	0	0	0	0
Kon u de verloskundige zorgverlener vragen stellen als u iets wilde weten?	0	0	0	0

62. Wilt u hieronder het cijfer **omcirkelen** dat het best uw **totaaloordeel** weergeeft over de zorg door uw verloskundige zorgverlener **tijdens de kraamperiode**?
1 betekent: de slechtst mogelijke zorg en 10 betekent: de best mogelijke zorg

De slechtst mogelijke zorg — De best mogelijke zorg

1 2 3 4 5 6 7 8 9 10

*Indien u professionele kraamzorg thuis heeft gehad, beantwoord dan graag de volgende vragen (**63 t/m 65**). Anders, ga door naar vraag **66**.*

63. Wilt u voor de volgende vragen aangeven hoe vaak dit **tijdens de kraamperiode** het geval was?

	Nooit	Soms	Meestal	Altijd
Had u het gevoel dat uw baby bij de kraamverzorgende in vertrouwde handen was?	0	0	0	0
Had u het gevoel dat u zelf bij de kraamverzorgende in vertrouwde handen was?	0	0	0	0
Legde de kraamverzorgende dingen op een begrijpelijke manier uit?	0	0	0	0
Behandelde de kraamverzorgende u beleefd?	0	0	0	0
Luisterde de kraamverzorgende aandachtig naar u?	0	0	0	0
Nam de kraamverzorgende u serieus?	0	0	0	0
Besteedde de kraamverzorgende voldoende tijd aan u?	0	0	0	0
Besteedde de kraamverzorgende voldoende tijd aan uw baby?	0	0	0	0
Besteedde de kraamverzorgende voldoende tijd aan de rest van het gezin?	0	0	0	0
Kon u de kraamverzorgende vragen stellen als u iets wilde weten?	0	0	0	0

*Indien u of een van uw ouders in het buitenland is geboren, beantwoord dan graag de volgende vraag (**64**).*

64.

	**Nooit**	**Soms**	**Meestal**	**Altijd**	**Niet van toepassing**
Had de kraamverzorgende begrip voor thuisgewoontes rond verzorging van de baby, die anders zijn dan bij autochtone Nederlanders?	0	0	0	0	0

65. Wilt u hieronder het cijfer omcirkelen dat het best uw totaaloordeel weergeeft over de zorg door de kraamverzorgende tijdens de kraamperiode?
1 betekent: de slechtst mogelijke zorg en 10 betekent: de best mogelijke zorg

De slechtst mogelijke zorg — De best mogelijke zorg
1 2 3 4 5 6 7 8 9 10

Mogelijk heeft u sinds de bevalling last gehad van enige gezondheidsproblemen. Hieronder volgt een aantal vragen over eventuele gezondheidsklachten en contacten met hulpverleners.

66. Heeft u **in de eerste drie weken na de geboorte** van uw kind klacht(en), ziekte(n) of verwonding(en) gehad, waardoor u het misschien rustiger aan moet doen? (Deze hoeven niet aan de zwangerschap gerelateerd te zijn)
(meerdere antwoorden mogelijk)
0 geen klachten/ziekten/verwondingen → *ga door naar vraag **68.***
0 rugpijn
0 bekken
0 knip/ingescheurd/hechtingen
0 (wond van) keizersnee
0 bloedverlies
0 depressiviteit/neerslachtigheid
0 anders, namelijk….

67. Heeft u hiervoor een **hulpverlener** geraadpleegd?
0 nee
0 ja → Welke hulpverleners heeft u hiervoor geraadpleegd? *(meerdere antwoorden mogelijk)*
0 verloskundige
0 huisarts
0 fysiotherapeut
0 anders, namelijk ………………………………………………………

68. Heeft u in de periode **vanaf drie weken na de bevalling tot nu** klacht(en), ziekte(n) of verwonding(en) gehad, waardoor u het misschien rustiger aan moet doen? (Deze hoeven niet aan de zwangerschap gerelateerd te zijn)
(meerdere antwoorden mogelijk)
0 geen klachten/ziekten/verwondingen → *ga door naar vraag **70.***
0 rugpijn
0 bekken
0 knip/ingescheurd/hechtingen
0 (wond van) keizersnee
0 bloedverlies
0 depressiviteit/neerslachtigheid
0 anders, namelijk….

69. Heeft u hiervoor een **hulpverlener** geraadpleegd?
0 nee
0 ja → Welke hulpverleners heeft u hiervoor geraadpleegd? *(meerdere antwoorden mogelijk)*
0 verloskundige
0 huisarts
0 fysiotherapeut
0 anders, namelijk ………………………………………………………

CONTACT MET ZORGVERLENERS

70. Kunt u aangeven of u, sinds het begin van uw zwangerschap, naast de verloskundige, ook **contact** gehad heeft met de volgende **zorgverleners**? Indien nee, geef dan bij het aantal contacten 0 aan.

**Zorgverlener**	**Aantal contacten/bezoeken**						
	**0**	**1-3**	**4-6**	**7-9**	**10-12**	**13-15**	**>15**
Gynaecoloog	0	0	0	0	0	0	0
Andere specialist[1]	0	0	0	0	0	0	0
Huisarts	0	0	0	0	0	0	0
Tandarts	0	0	0	0	0	0	0
Fysiotherapeut	0	0	0	0	0	0	0
Mensendieck therapeut	0	0	0	0	0	0	0
Cesartherapeut	0	0	0	0	0	0	0
Mondhygiëniste	0	0	0	0	0	0	0
Logopedist	0	0	0	0	0	0	0
Ergotherapeut	0	0	0	0	0	0	0
Podotherapeut	0	0	0	0	0	0	0
Orthoptist	0	0	0	0	0	0	0
Diëtist	0	0	0	0	0	0	0
Lactatiekundige	0	0	0	0	0	0	0
Wijkverpleegkundige	0	0	0	0	0	0	0
Kraamzorg	0	0	0	0	0	0	0
Gezinsverzorgende (voor hulp bij huishoudelijk werk)	0	0	0	0	0	0	0
Gezinsverzorgende (voor hulp bij dagelijkse verzorging)	0	0	0	0	0	0	0
Andere zorgverlener[2]	0	0	0	0	0	0	0

[1] *Indien u contact heeft gehad met een andere specialist, beantwoord dan graag de volgende vraag (**71**).*

71. Wat voor **specialist**(en) was(waren) dit? ………………………………………..

[2] *Indien u contact heeft gehad met een andere zorgverlener, beantwoord dan graag de volgende vraag (**72**).*

72. Wat voor **zorgverlener**(s) was(waren) dit? ……………………………………

73. Heeft u sinds uw bevalling in een **ziekenhuis** gelegen?
0 nee
0 ja ⟶ Wat was de reden van opname in het ziekenhuis?
……………………………………………………………………………………………………

74. Heeft u sinds het begin van uw zwangerschap **contact** gehad met onderstaande **psychosociale** hulpverleners en/of instellingen? Indien nee, geef dan bij het aantal contacten 0 aan.

**Psychosociale hulpverlener / instelling**	**Aantal contacten**						
	**0**	**1-3**	**4-6**	**7-9**	**10-12**	**13-15**	**>15**
RIAGG	0	0	0	0	0	0	0
Vrij gevestigd psycholoog	0	0	0	0	0	0	0
Vrij gevestigd psychiater	0	0	0	0	0	0	0
Vrij gevestigd psychotherapeut (geen psychiater)	0	0	0	0	0	0	0
Polikliniek psychiatrie	0	0	0	0	0	0	0
Consultatiebureau voor Alcohol en Drugs (CAD)	0	0	0	0	0	0	0
Seksuoloog	0	0	0	0	0	0	0
Maatschappelijk werker	0	0	0	0	0	0	0
Andere psychosociaal hulpverlener[1]	0	0	0	0	0	0	0

[1] *Indien u contact heeft gehad met een <u>andere psychosociaal</u> hulpverlener, beantwoord dan graag de volgende vraag (**75**).*

75. Wat voor andere **psychosociaal hulpverlener**(s) was(waren) dit? …………………………………….

76. Heeft u sinds het begin van uw zwangerschap voor uzelf **contact** gehad met een of meer van volgende **alternatieve behandelaars**? Indien nee, geef dan bij het aantal contacten 0 aan.

**Alternatieve behandelaar**	**Aantal contacten**						
	**0**	**1-3**	**4-6**	**7-9**	**10-12**	**13-15**	**>15**
Acupuncturist	0	0	0	0	0	0	0
Antroposofisch behandelaar	0	0	0	0	0	0	0
Homeopaat (niet eigen huisarts)	0	0	0	0	0	0	0
Manueel behandelaar (chiropractie, osteopathie, manuele therapie)	0	0	0	0	0	0	0
Natuurgenezer (voedingstherapie, neuraal therapie, kruidentherapie)	0	0	0	0	0	0	0
Paranormaal behandelaar (helderziende, gebedsgenezer, magnetiseur)	0	0	0	0	0	0	0
Andere alternatief behandelaar[1]	0	0	0	0	0	0	0

[1] *Indien u contact heeft gehad met een <u>andere alternatief behandelaar</u>, beantwoord dan graag de volgende vraag (**77**).*

77. Wat voor **alternatief behandelaar**(s) was(waren) dit? …………………………………..

78. Met welke **andere zorgverleners of instellingen** heeft u **contact** gehad sinds het begin van uw zwangerschap? Indien geen contact, geef dan bij het aantal contacten 0 aan.

**Zorgverlener / Instelling**	**Aantal contacten**						
	**0**	**1-3**	**4-6**	**7-9**	**10-12**	**13-15**	**>15**
Thuiszorgwinkel	0	0	0	0	0	0	0
Geestelijk verzorger	0	0	0	0	0	0	0
Consultatiebureau	0	0	0	0	0	0	0
Bedrijfsarts	0	0	0	0	0	0	0
Gemeentelijke gezondheidsdienst (GGD)	0	0	0	0	0	0	0
Zwangerschapscursus	0	0	0	0	0	0	0
Andere zorgverlener/instelling[1]	0	0	0	0	0	0	0

[1] *Indien u contact heeft gehad met een andere zorgverlener/instelling, beantwoord dan graag de volgende vraag (**79**).*

79. Wat voor **zorgverlener(s)/instelling**(en) was(waren) dit? …………………………………

*Indien u of een van uw ouders in het buitenland is geboren, beantwoord dan graag de volgende vraag (**80**).*

80. Heeft u voor deze zwangerschap gebruik gemaakt van de zorg door een verloskundige en/of gynaecoloog in het land waar u of uw ouders geboren zijn?
(meerdere antwoorden mogelijk)
0 ja, voor kinderwens
0 ja, voor controles
0 ja, voor ……………………………………………………………………..
0 nee

GEZONDHEID EN GEZONDHEIDSGEDRAG

De zwangerschap en bevalling hebben waarschijnlijk een behoorlijke invloed op je lichaam gehad. De zorg voor een (extra) kind kan uw gezondheid mogelijk ook beïnvloeden. We stellen nu een aantal vragen over uw gezondheid en hoe u zich voelt (als u al eens eerder voor dit onderzoek benaderd bent kan het voorkomen dat sommige vragen nogmaals worden gesteld omdat uw gevoelens, gezondheid en gedrag kunnen veranderen over de tijd).

81. Hoe zou u, over het algemeen genomen, uw **gezondheid** op dit moment omschrijven?
0 uitstekend
0 zeer goed
0 goed
0 matig
0 slecht

82. Hoe zou u, alles bij elkaar genomen, uw **functioneren** in huis, op het werk en in uw vrije tijd kunnen omschrijven?
0 uitstekend
0 zeer goed
0 goed
0 matig
0 slecht

83. Hoe zou u, over het algemeen genomen, de **gezondheid van uw baby** op dit moment omschrijven?
0 uitstekend
0 zeer goed
0 goed
0 matig
0 slecht

84. Hoeveel uur **slaapt u op dit moment** meestal per etmaal?
…………… uur

85. Hoeveel uur **sliep** u meestal per etmaal in de weken **vóór de bevalling**?
…………… uur

86. Kunt u aangeven hoe goed of slecht u sinds de bevalling slaapt?
0 heel goed ⟶ *ga door naar vraag **88.***
0 goed ⟶ *ga door naar vraag **88.***
0 niet goed, niet slecht
0 slecht
0 heel slecht

87. **Waarom** slaapt u niet zo goed? *(meerdere antwoorden mogelijk)*
0 ik kan niet makkelijk inslapen
0 ik kan niet makkelijk doorslapen
0 ik wordt vaak wakker van de baby
0 ik moet vaak voeden
0 anders, namelijk ……………………………………………..

88. Geeft u uw baby **borstvoeding**?
0 ja, alleen borstvoeding ⟶ *ga door naar vraag **90.***
0 ja, gecombineerd met flesvoeding ⟶ *ga door naar **90.***
0 nee, eerste dagen/weken wel, maar nu niet meer
0 nee, helemaal niet aan begonnen

89. Wat is de belangrijkste **reden** dat u geen borstvoeding (meer) geeft? *(**1** antwoord geven)*
0 slechte ervaring met borstvoeding
0 flesvoeding is makkelijker
0 bij flesvoeding kan de partner ook de baby eten geven
0 ik denk dat het slecht is voor (de vorm van) mijn borsten
0 borstvoeding is vermoeiend
0 borstvoeding doet pijn
0 mijn baby dronk niet goed
0 borstvoeding is moeilijk te combineren met mijn werk
0 medische reden
0 anders, namelijk ………………………………………….

90. Hoe staat het op dit moment met uw **vermoeidheid**?
0 Ik ben niet vermoeid
0 Ik ben enigszins vermoeid
0 Ik ben erg vermoeid

91. Hoe staat het op dit moment met uw **mobiliteit**?
0 Ik heb geen problemen met lopen
0 Ik heb enige problemen met lopen
0 Ik ben bedlegerig

92. Hoe staat het er op dit moment voor met de **zorg voor uzelf**?
0 Ik heb geen problemen om mijzelf te wassen of aan te kleden
0 Ik heb enige problemen om mijzelf te wassen of aan te kleden
0 Ik ben niet in staat mijzelf te wassen of aan te kleden

93. Hoe staat het er op dit moment voor met uw **dagelijkse activiteiten**?
0 Ik heb geen problemen met mijn dagelijkse activiteiten
0 Ik heb enige problemen met mijn dagelijkse activiteiten
0 Ik ben niet in staat mijn dagelijkse activiteiten uit te voeren

94. En in hoeverre heeft u momenteel **pijn en andere klachten**?
0 Ik heb geen pijn of andere klachten
0 Ik heb matige pijn of andere klachten
0 Ik heb zeer ernstige pijn of andere klachten

95. Hoe staat het er momenteel voor met uw **stemmingen**?
0 Ik ben niet angstig of somber
0 Ik ben matig angstig of somber
0 Ik ben erg angstig of somber

96. Heeft u last van één of meer langdurige **ziekten, aandoeningen of handicaps**?
0 nee
0 ja, ik het last van *(meerdere antwoorden mogelijk):*
0 allergie
0 astma
0 bekkenklachten
0 rugklachten
0 whiplash
0 anders, namelijk …………………………………………………………………………..….

97. In de afgelopen vier weken, op gemiddeld hoeveel dagen per week bent u, alles bij elkaar opgeteld, tenminste een half uur bezig geweest met **fietsen, klussen, tuinieren of sporten**?
……………..… dagen.

98. Dit moet mijn verloskundige vooral blijven doen:
……………………………………………………………………………………………………..
……………………………………………………………………………………………………..

99. Dit zou mijn verloskundige beter anders kunnen doen:
……………………………………………………………………………………………………..
……………………………………………………………………………………………………..

100. Wilt u nog opmerkingen of aanvullingen op deze vragenlijst geven, dan kunt u die hier opschrijven.
……………………………………………………………………………………………………..
……………………………………………………………………………………………………..
……………………………………………………………………………………………………..

HARTELIJK DANK VOOR HET INVULLEN VAN DE VRAGENLIJST
